# Systems Thinking About SARS-CoV-2

**DOI:** 10.3389/fpubh.2020.585229

**Published:** 2020-10-28

**Authors:** Rainer Johannes Klement

**Affiliations:** Department of Radiation Oncology, Leopoldina Hospital, Schweinfurt, Germany

**Keywords:** COVID-19, interdisciplinarity, public health, systemism, transdisciplinarity

## Introduction: Systemism and Systems Thinking

“[T]he good physician is a systemist: she prefers syndromes to isolated symptoms, places the body in its environment, and takes into account all the relevant levels of organization of matter, from the physical to the social.” (1, pp.45-46).

This quote from Mario Bunge, the Argentinian physicist and philosopher who authored more than 500 papers and 50 books and died at age 100 this year, will be taken as the starting point for a critical appraisal of the role that systems thinking has played or not in the management of the SARS-CoV-2 epidemic.

The terms “systemist” in Bunge's quote and “systems thinking” are closely connected; the former relates to ontology, the latter to a corresponding epistemology. According to Bunge, a systemist is someone who is committed to the worldview of systemism which immune system, can be summarized in the formula “Every existent is either a system or part of a system” [(1), p.47]. A consequence of systemism is that the generation of knowledge about the world requires the usage of certain analytic skills in order to identify and understand systems, predict their behavior and modify them in order to produce desired effects (experimentation). This epistemological approach will be defined as systems thinking (2). Accordingly, a good physician should be a systems thinker, someone who tries to identify and take into account the various systems and their components that make up and interact with a given patient. The skills required for systems thinking consist of recognizing interconnections between parts of a system (the base level of systems thinking), identifying and understanding cause-effect feedback loops, understanding system structure, dynamic behavior and systems at different scales (“systems of systems”), and lessening a system's complexity through various methods such as reduction or abstraction (2). These analytic skills are not only important when dealing with an individual patient, but especially when the aim is to improve population health through cross-disciplinary research, i.e., multi-, inter-, and transdisciplinarity (3, 4). Thereby, according to the definition of Rosenfield (3), multidisciplinarity means that researchers from several subdisciplines independently tackle a research problem in parallel or sequentially, i.e., without really working together, to contribute to an overall picture or solution. Interdisciplinarity also involves researchers working within their specific subdisciplines, but now jointly together. Finally, transdisciplinarity transcends disciplinary borders by working in a shared conceptual framework. Transdisciplinarity requires cross-disciplinary understanding between members of the research team and is necessary to obtain knowledge about emergent phenomena within systems [(4), p.86]. Such emergent phenomena cannot be explained by referring to lower levels of a system, i.e., via reduction. Transdisciplinary research and knowledge is therefore especially relevant for public health problems which involve emergent phenomena (5).

## Implications of Systems Thinking During the SARS-CoV-2 Outbreak

Unfortunately, we live in an age in which fewer and fewer scholars have serious competence beyond their own increasingly narrow field of research (6). This is particularly reflected within the medical sciences, in which material reductionism, the view that every level of phenomena can be explained by causal effects of material particles at a lower level, is the default ontology (7, 8), apparently superseding cross-disciplinary, and in particular transdisciplinary research. While material reductionism has led to great advances in the natural sciences dealing with the non-living world, it faces serious problems when applied to sciences dealing with living, multicellular organisms and their societies both of which can be conceptualized as open systems with emergent properties (9, 10). Thus, physicians and public health authorities should resist reductionist thinking and instead try to identify and study system structures and causal loops of the problem at hand, integrating all relevant disciplines within an inter- and transdisciplinary approach.

**Figure 1:**
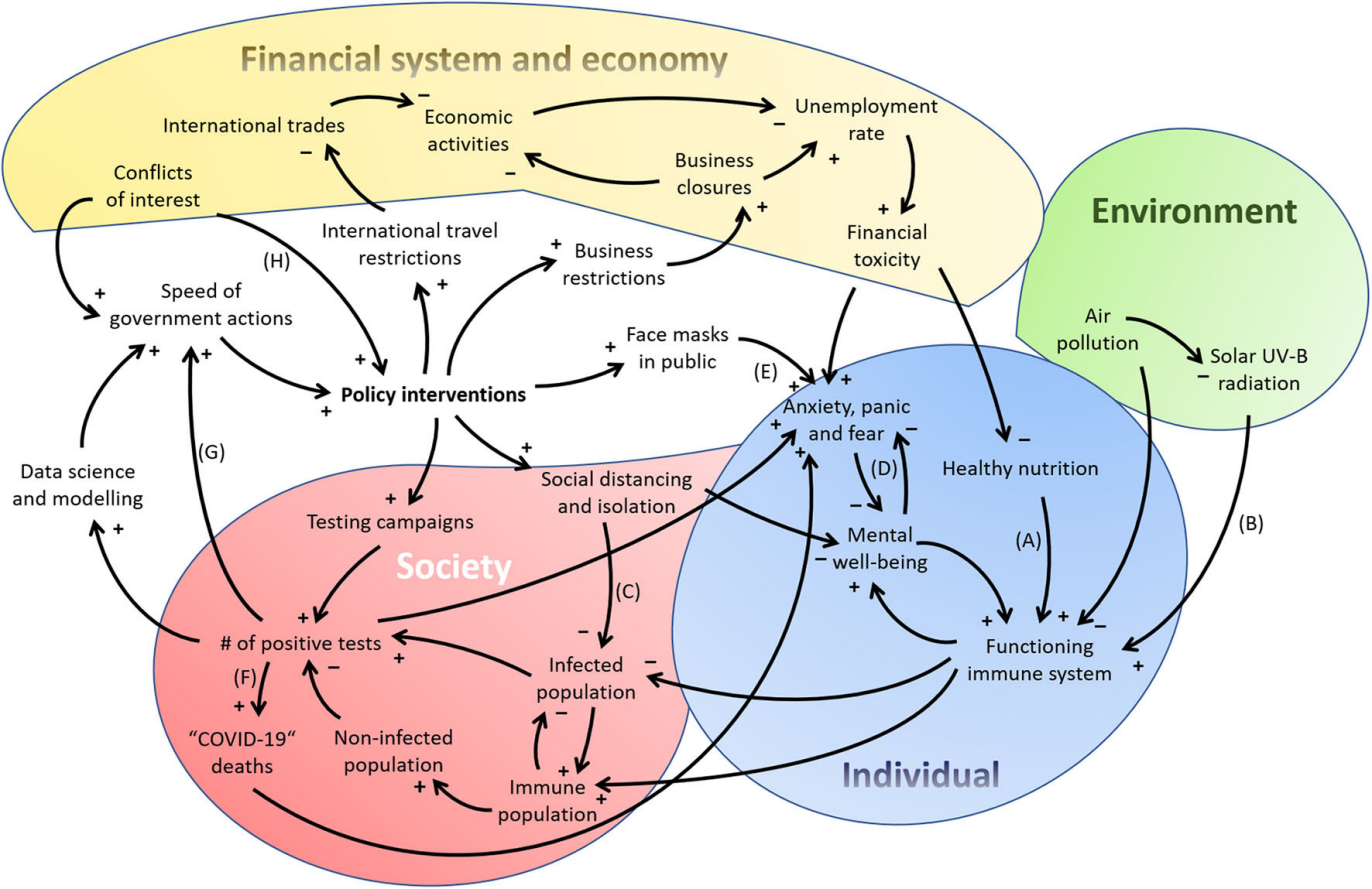
A preliminary causal loop diagram displaying the complexity of the SARS-CoV-2 crisis' environmental-individual-socio-economic-political system. Based on Figure 1 of Sahin et al. (11), but extended in some components and simplified in others not relevant for my main arguments here.

Sahin et al. (11) recently developed a preliminary causal loop diagram (CLD) depicting many of the causal feedback loops within the environmental-health-socio-economic system of the SARS-CoV-2 problem. While their CLD is a valuable starting point for informing policy interventions against the SARS-CoV-2 and future outbreaks of other infectious pathogens, it has neglected the system of the individual person that the various medical disciplines are concerned with. I have therefore created a modified CLD based on the work of Sahin et al. (11) which includes the system of an individual and other components that I found to be under-represented in discussions about the SARS-CoV-2 crisis (Figure 1). These are briefly described in the following.

### A Functioning Immune System

A functioning immune system on the level of the individual is a major determinant of the susceptibility to infection as well as the severity of symptoms. The fact that ~40–45% of SARS-CoV-2 infections remain asymptomatic (12) indicates that innate and adaptive immunity have the power to effectively handle this virus. For example, triggering of innate immune adaptions through influenza vaccination has been proposed as a protective measure against COVID-19 severity (13), which indeed received some confirmation in (not yet peer-reviewed) observational studies (14, 15). It has also been proposed that the adaptive immune system may account for a significant protection in certain individuals through cross-reactivity between B- and T-cell epitopes of SARS-CoV-2 and other human coronaviruses (16). Indeed, cross-reactive T-cell responses against SARS-CoV-2 associated with “common cold” coronaviruses have been detected in about 30–80% of unexposed individuals (17–20). Unfortunately, the computer models that had been used to justify the lockdown interventions in many Western countries had not taken these immune responses offering partial protection of a significant percentage of the population into account (21, 22)^1^–an example of “looking at only one or a few dimensions of the problem at hand” (reductionism) and “lack of expertise in crucial disciplines” (inter- and transdisciplinarity) that Ioannidis et al. identified as factors contributing to these models wrongly predicting COVID-19 fatalities by orders of magnitude (24).

Furthermore, the public is rarely informed that an optimally functioning immune system requires the presence or absence of certain factors. Some of these factors are studied within the transdisciplinary field of *nutritional immunology*. Healthy nutrition, i.e., an optimal macro-, micro- and trace nutrient composition, positively supports innate and adaptive immunity (path A in Figure 1). While the interactions between nutrition and the immune system are complex and pose a multidimensional problem (25), it is well-established that an adequate intake of protein and certain vitamins and trace elements is needed for an optimally functioning immune system and the containment of respiratory virus-induced inflammation (26, 27). For example, SARS-CoV-2, influenza and other respiratory viruses activate the cytoplasmic nucleotide-binding domain (NOD)-like receptor protein 3 (NLRP3) inflammasome in immune cells (typically monocytes and macrophages), which produces and activates interleukin (IL)-1β and further downstream cytokines, causing flu-like symptoms and tissue damage (28). Several nutrients and secondary plant substances have been shown to reduce NLRP3 inflammasome activation (29), among them vitamin C (ascorbic acid), which may be especially active against coronaviruses (30), or the ketone body β-hydroxybutyrate (31). Evidence for protective effects against COVID-19 has emerged for some of these nutrients, in particular zinc, selenium, N-acetyl-cystein and vitamin C, although it is limited to non-randomized studies (27, 32). The strongest evidence to date is available for vitamin D whose main natural supply is not through diet, but solar UV-B radiation on the skin (path B in Figure 1). Higher vitamin D levels have been linked to lower COVID-19 incidence, death rates and hospitalizations in epidemiological studies (33–35). First clinical data suggested that higher vitamin D levels are associated with less severe courses of COVID-19 (36, 37). A recent randomized controlled trial has shown a highly significant benefit of high vitamin D supplementation in COVID-19 patients: out of 50 patients receiving 25-hydroxyvitamin D in addition to standard treatment^2^ only one required intensive care unit admission compared to 13 out of 26 patients having not received vitamin D (*p* < 0.001) (38). Given the cost-effectiveness and safety of vitamin D and other immune-supporting nutrient supplements, some authors have rightfully argued that public health officials should encourage their adequate intake through a healthy diet and supplementation (26, 27).

Other important insights into immune system regulation come from the field of *psychoneuroimmunology*. This field investigates how psychological stress disrupts hormone and immune regulation; stress in mice, e.g., increases IL-1β through NLRP3 activation in the hippocampus (39). Mario Bunge goes even further by claiming that stress crosses not only three, but five disciplinary boundaries. He includes in this consideration “all levels of organization,” up to the social, making stress a “psycho-neuro-endocrino-immuno-social disease” [(1), p.68]. Social distancing and isolation, while possibly decreasing the transmission of infectious pathogens (path C in Figure 1), also decreases mental well-being by increasing psychological stress, anxiety and fear (40–42) (loop D in Figure 1). Enforced prolonged wearing of face masks is also problematic, as demonstrated by Daniela Prousa who revealed that ~60% of the German population experienced severe psychosocial problems already 5–7 weeks after installment of a public mask wearing decree (43) (path E in Figure 1).

### SARS-CoV-2 Tests and Statistical Illiteracy

Testing for SARS-CoV-2 using polymerase chain reaction (PCR) or serum antibody tests is required to accurately map the spread of the disease within and across nations, although politics have failed to use such data in international cooperation (44). Furthermore, efforts to obtain reliable estimates for test sensitivity, specificity and the so-called base rate (or disease prevalence) have been sparse, although these quantities are essential for the logical inferences that can be made from a positive test result (45).

Some studies reported problems with both sensitivity and specificity of commercially available SARS-CoV-2 PCR tests (46, 47). Instead of acknowledging these limitations, positively tested individuals are still routinely nominated as infected individuals in the media, which is *de facto* wrong. Furthermore, many newspapers still simply report the daily or cumulative amount of positive PCR tests, without standardizing to the total number of tests performed and/or population number. This could lead to the impression that the prevalence of SARS-CoV-2 infection rises even if it declines or stays constant. For example, in Germany the number of weekly SARS-CoV-2 PCR tests has been increased to over one million until the end of August 2020, so that the absolute number of positive tests increased along with the number of performed tests, while the percentage of positive tests had remained <1.5% since mid-May and ≤1.0% since end of June (48). Still, German chancellor Angela Merkel proclaimed in a press conference on August 28th that “the infection numbers have clearly risen during the past weeks” (49). Furthermore, in their discussion of the test statistics, even the Robert-Koch-Institute did not mention that the base rate needs to be accounted for when interpreting a positive test result (48), thereby committing what is called the base rate fallacy (50).

The “collective statistical illiteracy” of health care professionals, journalists and politicians (51, 52) is nothing more than a lack of transdisciplinary knowledge in mathematics and statistics. It is contributing to incorrect information about the spread of the SARS-CoV-2 with the effect of increasing both the public fear and impulsive actions from governments (paths F and G in Figure 1).

### Learning From Past Epidemics

Given the leading role of the World Health Organization (WHO) in estimating the severity of infectious disease outbreaks, we should consider how the WHO has influenced policy decisions in the past. Doing so, it appears that the WHO has overestimated the severity of several recent “pandemics”: SARS in 2002/2003, avian flu in 2005/2006 and Swine flu in 2009. This was likely due to the WHO basing its recommendations on a reductionist assessment made by molecular virologists (53), a mistake that I think is repeated in the current SARS-CoV-2 epidemic.

In addition, financial ties with the pharmaceutical industry of scientific advisors to WHO and international and national public health institutions have likely influenced public health policies during past virus outbreaks, e.g., driving a massive vaccination campaign during the swine flu pandemic that earned the pharmaceutical industry 18 billion Euro (53). Today, the WHO is financed to a large degree by the private Bill and Melinda Gates foundation from which it received more than 228 million US$ in 2018 (54). The Bill and Melinda Gates foundation also funds several institutes that have large influence on decision-makers during the COVID-19 epidemic^3^, as well as the GAVI vaccine alliance which in turn funds the WHO (55, 56). Learning from past epidemics means that critical journalists and scientists must watch carefully if financial conflicts of interest might again influence policy decisions during the SARS-CoV-2 crisis (path H in Figure 1), in particular if these decisions cannot be justified by inter- and transdisciplinary science.

## Discussion

The complexity of the SARS-CoV-2 crisis, and most of the cross-disciplinary considerations associated with it, should have profound consequences for public health measures and personal behavior (57). If the system of an individual is considered, it must be asked why policies have not been directed more toward a positive message of self-responsibility in the sense that people can actively strengthen their immune system. Instead, the daily media messages about the latest rise in infection numbers (which as stated above are only positively tested persons) as well as the installment of drastic measures all over the World fuel the narrative of us all being potential victims of a killer virus that can only be held back through physical barriers, extreme hygiene and ultimately vaccination (58)—a reductionist approach purely focused on the virus without considering the context of the human host, its immune system, microbiome and economic, social and natural environment. This raises many severe problems. For example, in poorer countries inadequate nutrition, financial toxicity and extreme stress induced by governmental lockdown measures without adequate relief strategies can lead to many deaths that remain invisible compared to those presented on COVID-19 dashboards; they can be attributed to a reductionist epidemiological and/or virological view of the problem (59). Along these lines, reductionist thinking raises many ethical issues, namely if avoiding risk of infection at any cost should outweigh other human values such as mental health, social contacts, dying in presence of the family, and basic human rights such as adequate nutrition and freedom of peaceful assembly. Here, more interdisciplinary discussions among health care professionals and scholars of the arts and humanities appear necessary.

As a final example, if systems thinking is employed it should be clear that the high death rates in Northern Italy in Spring 2020 could not simply be extrapolated to other countries given the characteristics of the population [very old, many smokers, high mesothelioma rates (60)], the environment (one of the most crowded and heaviest air-polluted regions in Italy) and the healthcare system [“decades of financial cuts, privatization, and deprivation of human and technical resources” (61)]. Although Italy has often been used for sustaining the mainstream “deadly virus” narrative for the public, such details about the healthcare system and population characteristics are specialist facts that make life more complicated, but need to be considered in order to avoid unnecessary public fear (58).

In summary, it is my argument that journalism, politics, and medicine involved within the SARS-CoV-2 crisis have maintained a rather simple narrative and reductionist thinking thus far. In my opinion we need more journalists interested in accurately informing the public about the complex facts associated with SARS-CoV-2; we further need more politicians willing to be advised from a much broader spectrum of industry- and financially independent scholars than just a few selected virologists and epidemiologists with putative financial or other conflicts of interest. Finally, we need more inter- and transdisciplinary science (62), in particular as retrospective analyses indicate that some drastic policy decisions had no clear benefit (63, 64), and may even have caused more harm than good (43, 59, 65). My hope is that the critical systems perspective on the COVID-19 crisis presented here may be considered for the improvement of both public health and individual well-being.

## Author Contributions

RJK conducted the research and wrote and approved the manuscript.

## Conflict of Interest

The author declares that the research was conducted in the absence of any commercial or financial relationships that could be construed as a potential conflict of interest.

## Footnotes

^1^I have thoroughly analyzed this and other problems in the modeling study of Flaxman et al. (22) in a German online article (23); for example, their model assumes that every infection causes a secondary infection until 100% of the population had been infected which is at odds with the observations of pre-existing immunity.

^2^The dose was 0.532 mg (21280 I.U.) on day 1, followed by 0.266 mg (10640 I.U.) on days 3 and 7 and 0.266 mg weekly thereafter (38).

^3^These include the Johns Hopkins University which maintains the global SARS-CoV-2 statistics, the German Robert Koch-Institute and the Charité Hospital in Berlin which employs Prof. Drosten, one of the leading government advisors during the crisis (55).
